# The Relationship between Living Arrangements and Sleep Quality in Older Adults: Gender Differences

**DOI:** 10.3390/ijerph19073893

**Published:** 2022-03-24

**Authors:** Hyeon Sik Chu, Juyeon Oh, Kounseok Lee

**Affiliations:** 1 College of Nursing, Hanyang University, Seoul 04763, Korea; healingchu@hanyang.ac.kr; 2 College of Nursing, Dankook University, Cheonan 31116, Korea; ohjy@dankook.ac.kr; 3 Department of Psychiatry, Hanyang University Medical Center, Seoul 04763, Korea

**Keywords:** older adults, sleep, gender differences, community health survey

## Abstract

(1) Background: This study examined the effects of living arrangements on the quality of sleep among older adults and analyzed related gender differences; (2) Methods: A total of 4756 older adults in Seoul were included. After adjusting for socio-demographic factors, older adults living alone showed a poorer sleep quality compared with those living with others; (3) Results: When we analyzed the effects of living arrangements on sleep quality by gender, there was no difference in the risk of poor sleep quality between male older adults living alone and those living with others (OR = 1.089, 95% CI = 0.729–1.628), whereas the risk of poor sleep quality was higher for female older adults living alone than those living with others (OR = 1.359, 95% CI = 1.088–1.696); (4) Conclusions: In this study, we have confirmed that older women living alone had poor sleep quality compared to older men. Hence, gender-based approaches will be helpful when providing social support resources to older adults living alone.

## 1. Introduction

Population aging is a rapidly accelerating global phenomenon, with the older population aged 65 and above estimated to be doubling to 1.5 billion by 2050 [1]. South Korea is one of the most rapidly aging countries; the proportion of adults over 65 years was 15.5% at the end of 2019 and is expected to exceed 20% by 2025, causing it to become a super-aging society [2]. Although adult children are expected to live with their aged parents and care for them based on traditional Korean beliefs, the living arrangements of older adults have changed rapidly over the years due to industrialization, urbanization, and weakening filial piety. In addition, population aging is significantly increasing the number of older adults living alone. For example, the proportion of extended families living with older adults decreased from 24.5% in 1985 to 6.2% in 2015, while older adults living alone increased from 16.9% to 32.9% [3]. Because the biggest support system for older adults is assumed to be family in Korea, older adults living alone receive less social support from family and are more likely to have poor mental health, life satisfaction, and well-being [4,5,6,7]. Therefore, additional attention and support are needed to improve the health of older adults living alone.

Other than living arrangements, sleep-related issues are common among older adults. Normative aging is related to increased time needed to fall asleep and decreased total sleep time; [7] therefore, the quality of sleep among older adults is poorer than that of younger and middle-aged adults. Poor sleep quality affects many aspects of well-being and health, such as cognitive function, dementia, and even survival. Thus, sleep problems have received increasing attention within the context of geriatric research. Sleep-related issues in older adults are associated with various factors, including demographics, lifestyle, and physical and mental health status.

Family environment is another factor associated with sleeping-related issues. Many studies have reported that people living alone have a poorer quality of sleep and shorter sleep duration daily compared with those who live with household members [8,9,10]. However, it is not clear whether the phenomenon of living alone is an influencing factor that worsens the quality of sleep, especially in older adults. This is because, in general, older adults living alone are older and have a low income, less education, and more health problems, all characteristics related to poorer sleep quality.

Therefore, this study aimed to examine whether only the demographic and health-related characteristics of older adults living alone cause poor sleep quality, or whether the experience of living alone also affects sleep quality. Although there are many pharmacological treatments for better sleep quality, these treatments include a risk of side effects and some long-term effects. For non-pharmacological treatments, identifying the mechanism of low sleep quality in older adults living alone will enable more efficient, personalized intervention planning. Further, sleep characteristics are different between genders. For example, compared with older men, older women take a longer time to fall asleep, have less sleep efficiency, more daytime sleepiness, and a 40% increased risk of developing insomnia [11,12]. In addition, living arrangements differentially impact older adults’ lives based on gender. For example, older men living alone are at an increased risk of being socially isolated, and older women living alone have a lower quality of life and more psychological distress [13,14,15]. Therefore, we further analyzed the impact of living alone on the quality of sleep by gender. Accordingly, the hypotheses of this study are as follows: (1) Controlling for demographic and health-related characteristics, it was hypothesized that the phenomenon of living alone will affect the quality of sleep; (2) it was hypothesized that the phenomenon of living alone will affect the quality of sleep of both male and female older adults.

## 2. Materials and Methods

### 2.1. Design

This study was an observational study with a cross-sectional approach.

### 2.2. Data and Participants

This study was a secondary analysis utilizing raw data from the 2018 Korean Community Health Survey (KCHS) for the Seoul region. The KCHS is an annual nationwide health survey that has been performed by the Korea Disease Control and Prevention Agency since 2008 to provide health statistics for the establishment of regional health plans and developing evidence-based disease prevention and health promotion programs [16]. Participants in the KCHS were people living in the community, not people living in institutions such as long-term care facilities or hospitals.

**Figure 1:**
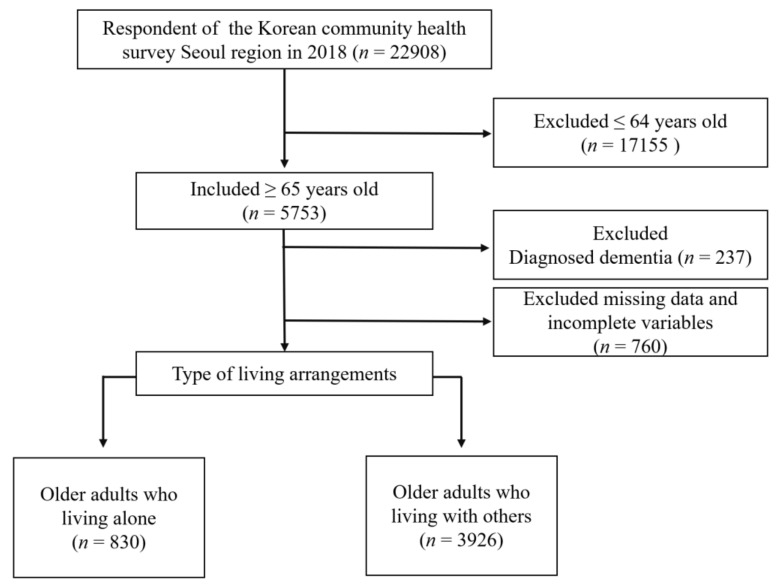
Flow chart of the study population.

The 2018 KCHS for the Seoul region was conducted between August and October 2018 and included a total of 22,908 household residents. Of those, 4756 were included in this present study, after excluding 18,152 participants because they were under 65 years of age, and/or were missing data on some study variables (Figure 1). When we excluded missing data and incomplete variables, we assumed that the missing data were missing completely at random [17]. In the 2018 KCHS, all the questionnaires were used by trained surveyors using the computer-assisted personal interview method. This study was waived for review by the Institutional Review Board (IRB) of Hanyang University (IRB No. HYUIRB-202108-013).

### 2.3. Measures

Sociodemographic variables included age, sex, marital status, educational attainment, monthly household income, living arrangement and employment status. Age was grouped into 65–74 years and 75 years and older and named “young elders” and “old elders”, respectively. Marital status was categorized as married or singled/divorced/bereaved. Educational attainment was categorized as illiteracy, less than elementary school, middle school, and more than high school graduation. The monthly household income of the participants was classified as less than USD 1000, under or less than USD 2000, less than USD 3000, and more than USD 3000. In the type of living arrangements, older adults living alone were defined as persons within a single-person household.

Health and health-related behavioral variables included self-rated health status, hypertension, diabetes mellitus (DM), body mass index (BMI), perceived stress, perceived cognitive decline, depressive symptoms, current smoking, current alcohol drinking, and regular exercise. Hypertension and DM were classified based on questions that asked whether participants had been diagnosed with each disease by a doctor or not. BMI was calculated as body weight (kg) divided by the square of height (m^2^). Body weight and height were measured by trained surveyors. Current smoking was assessed by asking about the amount of smoking per day or month and classified as a smoker (≥1 cigarette per month) and non-smoker (including ex-smoker) over the last year. Regular exercise was classified as active (regularly walking for more than 150 min per week) and inactive.

#### 2.3.1. The Patient Health Questionnaire (PHQ-9)

Depressive symptoms were measured using the Korean version of the Patient Health Questionnaire (PHQ-9), which is based on the Diagnostic and Statistical Manual of Mental Disorders, 4th edition (DSM-IV) diagnostic criteria for depressive disorders, namely anhedonia, depressed mood, trouble sleeping, feeling tired, change in appetite, feelings of guilt or worthlessness, trouble concentrating, feeling slowed down or restless, and suicidal thoughts. Each item is rated on a Likert scale from 0 (Not at all) to 3 (Nearly every day), and responses are summed to form a total score ranging from 0 to 27; higher scores indicate a higher level of depressive symptoms [18]. A score of 5 or above was used as a cut-off point when screening for Korean older adults with depressive disorder [19].

#### 2.3.2. Pittsburgh Sleep Quality Index (PSQI)

Sleep quality was measured using the Korean version of the Pittsburgh Sleep Quality Index (PSQI). PSQI is a standardized, self-administered questionnaire developed by Daniel J. Buysse that evaluates retrospective sleep quality and disturbances within the past month [20]. The PSQI-K consists of the same items as the original instrument; it comprises 19 items forming seven subscales: (1) sleep quality (1 item), (2) sleep latency (2 items), (3) sleep duration (1 item), (4) sleep efficiency (3 items), (5) sleep disturbance (9 items), (6) sleep medication (1 item), and (7) daily dysfunction (2 items). The PSQI-K was evaluated following the original scoring system [21]. Each component has a score that ranges from 0 to 3. The scores of seven components are summed to yield a PSQI global score ranging from 0 to 21. Respondents with a global score greater than 5 are classified as “poor sleepers”, while those with a score of 5 or less are classified as “good sleepers”. The original PSQI had an internal consistency of 0.83; in this study, internal consistency was 0.70.

### 2.4. Procedures

After obtaining IRB approval for the study procedures, we downloaded the 2018 KCHS Seoul region raw data on the KCHS website (https://chs.kdca.go.kr, accessed on 18 August 2021). Downloaded data was cleaned and analyzed by H.S.C. and the results were interpreted by O.J. and K.L.

### 2.5. Data Analysis

The collected data were analyzed using IBM SPSS Statistics 23.0 for Windows (Armonk, NY, USA) in consideration of the complex sampling design, and weighting was applied for population estimates. Descriptive statistics were calculated for each variable using frequencies, means, and standard deviation (SD) for categorical and continuous variables, respectively. The Rao–Scott chi-squared test and independent *t*-test were used to verify differences in the distributions of independent variables related to sleep quality according to living arrangements. Multivariate logistic regression analysis was performed to verify the effects of living arrangements on sleep quality by gender. All *p*-values were obtained using two-tailed tests, and *p* < 0.05 was considered statistically significant.

## 3. Results

### 3.1. Differences in Sociodemographic and Health-Related Characteristics According to the Type of Living Arrangement

Out of 4756 participants of this study, 830 (17.5%) and 3926 (82.5%) older adults live alone and with others, respectively. By analyzing the differences in sociodemographic and health-related characteristics between the groups of older adults living alone and with others (Table 1), this study found significant differences in all variables except DM, BMI, and perceived stress. The mean age of older adults living with others was 73.32 ± 6.17 years while that of older adults living alone was 74.92 ± 6.70 years, which is significantly higher (*t* = 6.342, *p* < 0.001). In terms of gender, the ratio of females was 49.9% and 78.9% in the groups who live with others and alone, respectively, which was a significant difference (Rao–Scott χ^2^ = 260.37, *p* < 0.001). As for monthly household income, 49.9% and 10.3% of older adults who live alone and with others had an income of less than $1000, respectively, which was a significant difference (Rao–Scott χ^2^ = 302.36, *p* < 0.001). In the case of self-rated health status, 39.0% of older adults living alone were evaluated as “unhealthy” which was significantly higher than that of the older adults living with others at 28% (Rao–Scott χ^2^ = 25.26, *p* < 0.001).

### 3.2. Differences in Sleep Quality According to the Type of Living Arrangement

Comparing sleep quality by the type of living arrangement (Table 2), the global PSQI score for older adults who live with others was 5.19 ± 3.38 compared with 6.47 ± 3.81 in older adults who live alone, which was significantly higher (*t* = 8.942, *p* < 0.001). The proportion of older adults who live with others who scored ≥6 on the global PSQI was 36.5% (1434), which was significantly lower than that of older adults who live alone (52.4%, 435) (Rao–Scott χ^2^ = 62.80, *p* < 0.001). Significant differences were observed in all subdomains of the PSQI, except for sleep quality and sleep disturbance, between older adults who live alone and those who live with others. Compared to older adults who live with others, older adults living alone had longer sleep latency, shorter sleep duration, lower sleep efficiency, and more use of sleeping pills and daytime dysfunction.

### 3.3. The Effects of Type of Living Arrangement on Sleep Quality

Before performing multivariate logistic analysis, we verified multicollinearity among the independent variables. All independent variables in this study had Variation Inflation Factor (VIF) values below 10 [22]. Therefore, we concluded that there was no multicollinearity.

To verify the effect of living arrangements on sleep quality, sociodemographic factors and health factors showing significant differences in sleep quality in the univariate analysis were selected as covariates.

The risk of poor sleep quality was higher for older adults living alone than for older adults living with others after adjusting additionally for age, gender, education level, household income, and employment status in model 1. The risk of poor sleep quality was significantly high among older adults living alone (odds ratio (OR) = 1.300, 95% confidence interval (CI) = 1.086–1.555). In model 2, which was model 1 with additional adjustments for current smoking, alcohol drinking, regular exercise, self-rated health status, perceived cognitive decline, hypertension, and depressive symptoms, the risk of poor sleep quality remained significantly higher among older adults living alone (OR = 1.276, 95% CI = 1.054–1.545; Table 3).

The risk of poor sleep quality within older adults living alone was analyzed for gender differences (Table 4). For both men and women, the risk of poor sleep quality was significantly higher for older adults living alone than for those living with others (OR = 1.595, 95% CI = 1.160–2.192 in men; OR = 1.542, 95% CI = 1.293–1.840 in women). In model 1, which was adjusted additionally for age, education level, household income, and employment status, there were significantly more poor sleepers among women than men among older adults living alone (OR = 1.353, 95% CI = 1.099–1.666 in women; OR = 1.208, 95% CI = 0.837–1.744 in men). In model 2, which was model 1 with additional adjustments for current smoking, alcohol drinking, regular exercise, self-rated health status, perceived cognitive decline, hypertension, and depressive symptoms, there was no difference in the risk of poor sleep quality for men between older adults living alone and those living with others (OR = 1.089, 95% CI = 0.729–1.628), whereas the risk of poor sleep quality for women was higher for older adults living alone than for older adults living with others (OR = 1.359, 95% CI = 1.088–1.696).

## 4. Discussion

This study was conducted to examine the effects of living alone according to gender on sleep quality and to empirically analyze whether poor sleep quality in older adults living alone was caused by sociodemographic and health-related characteristics or was a phenomenon caused by living alone.

In this study, the ratio of older adults living alone was 17.5%. Compared with other countries, this ratio is higher among Asian countries [23]. Research on the health of seniors living alone is urgently needed at this time because Korea is about to become a super-aged society. Additionally, population aging is rapidly progressing in this country.

The results indicate that older age, lower education, and lower monthly household income were significantly associated with older adults living alone. These findings are consistent with existing literature [24]. The representative and strong risk factor of “older age” is characteristic of individuals who live alone. This is accompanied by lower educational attainment and a high proportion of women [5]. In general, these sociodemographic characteristics are known to affect health-related characteristics. In this study, self-rated health status, the prevalence of hypertension, perceived cognitive decline, depressive symptoms, regular exercise, and current smoking and drinking showed a significant difference between older adults living alone and those living with others. Additionally, low self-rated health status, high prevalence of hypertension, perceived cognitive decline, and depressive symptoms were shown in older adults living alone. This result is like that of previous studies that have assessed older adults living alone [25,26,27,28]. Weissman and Russell analyzed the National Health Interview Survey of the United States Centers for Disease Control and Prevention and found that older adults living alone have a low self-rated health status, low ability to perform daily activities, and the prevalence of many chronic diseases [29]. However, a slight inconsistency in the results was shown in their health promotion activities. There was lower regular exercise performance in the group of older adults living alone than in the group living with others [30], but smoking and drinking were found to be higher in the group of older adults living with others than in those living alone. This is believed to have been caused by the high ratio of males in the group of older adults living with others [31]. Overall, it can be said that the health-related characteristics of older adults living alone align with this study as having a higher risk of self-neglect that threatens health and safety compared with older adults living with others [32].

This study employed multiple logistic regression analyses and found that sleep quality was significantly lower in older adults living alone, even after adjusting the sociodemographic and health-related factors. This agrees with the results of previous studies that have demonstrated that living alone has a negative effect on sleep quality [8]. Many studies have reported that the social isolation and loneliness experienced by older adults living alone has a higher negative effect on sleep quality than in those living with others [9,10]. There was a similar result in the study by Choi et al. [33], which demonstrated a significant difference in the PSQI global score and sleep disturbance among the PSQI subdomains in older adults living alone. Arber et al. have reported that persons who are divorced or widowed have significantly lower sleep quality [34]. As shown in the study results, one of the strongest sources of support in old age is a spouse, which may bring about healthier lifestyles, emotional support, the better maintenance of current life, and a better ability to cope with worsening health conditions than in those without a spouse [35]. Furthermore, living with a spouse or family was found to have a very positive correlation with mental health in old age, which can play the role of mutual support.

In addition, age was also significant as a covariate, which supports the findings of previous studies that an increase in age is associated with lower sleep quality [36]. The association between lower education level and poor sleep quality is also consistent with previous research results [37,38]. This is probably because a lower education level is associated with a lower socioeconomic level [39].

Interestingly, the difference in sleep quality according to living status was not statistically significant in older men after adjusting for sociodemographic factors. In other words, we found that sociodemographic factors affect sleep quality in older men, whereas health factors and living alone did not.

On the contrary, in older women, living alone had a significant negative effect on sleep quality, even after adjusting for sociodemographic and health-related characteristics. Although changes in hormones among women caused by puberty and menopause along with aging negatively affect sleep quality [40], this study found that living arrangements had an additional effect on sleep quality in older women. One of the reasons for the result that living alone had particularly affected the sleep quality of women, but not that of men, might be that psychosocial factors such as loneliness are frequently caused by living alone [41]. Despite difficulty in ascertaining this causality, a previous study also found that, among general adults, in men sleep quality is more affected by lifestyle factors such as socio-demographic factors, whereas psychosocial factors greatly affect sleep quality among women [42]. Therefore, differential approaches for improving sleep quality in older adults should be used according to gender.

In the same vein, more psychological distress of older women living alone could be associated with social isolation and decreased social support. Participation in social activities and social connectedness has a positive effect on physical and psychosocial health as well as sleep quality improvement. [9] In situations like the recent COVID-19 pandemic, older adults living alone are a group more vulnerable to social isolation [43]. In a situation where face-to-face interaction is practically difficult due to social distancing, participation in social gatherings and activities using non-face-to-face technologies via smartphones can be an effective support system for older adults living alone, and this may help improve their sleep quality [44]. Therefore, interventions that target social isolation might improve health status, including sleep quality and depressive symptoms, in older women living alone.

This study has several limitations. First, it is a secondary analysis of the 2018 community survey in Seoul, so its results are limited to generalizations of the entire older adults group. Moreover, this study employed a cross-sectional design, so it was difficult to infer causality. Second, in this study only the presence or absence of living alone was used as a research variable; however, variables such as the duration of living alone, social isolation, or participation in social gatherings were not included. Third, we only measured the quality of sleep through self-reporting and could not consider the sleep environment, such as the level of noise and light. Finally, we only classified types of living arrangements on living alone or living with others, but did not specify the type of cohabitant. In future research, it is necessary to explore the factors that influence the quality of sleep. Further, the use of objective measurement methods and various factors is also necessary.

Nevertheless, this study investigated the quality of sleep according to living arrangements for older adults living in urban areas and identified factors affecting sleep quality according to gender. Sleep quality is an important factor in maintaining physical and mental health among older adults. Identifying factors that affect their sleep quality can be used as important data for the development of interventions and programs to improve sleep quality.

## 5. Conclusions

In this study, we have confirmed that older women living alone had poor sleep quality compared to older men. Hence, gender-based approaches will be helpful when providing social support resources to older adults living alone. In particular, it is necessary to evaluate the quality of sleep in older women living alone.

## Figures and Tables

**Table 1:** Descriptive analysis of demographics and study variables (*n* = 4756).

Characteristics	Categories	Total	Living Alone (*n* = 830, 17.5%)	Living with Others (*n* = 3926, 82.5%)	Rao Scott χ^2^ or *t* (*p*)
		*n* (%) or M ± S.E.	*n* (%) or M ± SD	*n* (%) or M ± SD	
Age	Age (years)	73.50 ± 0.11	74.92 ± 6.70	73.32 ± 6.17	6.342 (<0.001)
	≥75 <75	3099 (66.0) 1657 (34.0)	473 (57.5) 357 (42.5)	2626 (67.6) 1300 (32.4)	28.53 (<0.001)
Gender	Male Female	2029 (45.6) 2727 (54.4)	157 (21.1) 673 (78.9)	1872 (50.1) 2054 (49.9)	260.37 (<0.001)
Marital status	Married Singled/Divorced/Bereaved	3225 (69.5) 1531 (30.5)	0 (0.0) 830 (100)	3225 (82.3) 701 (17.7)	1823.55 (<0.001)
Education status	Illiteracy ≤Elementary school Middle school ≥High school	406 (7.5) 1603 (32.3) 833 (18.0) 1914 (42.2)	119 (13.3) 343 (30.9) 127 (18.3) 241 (44.5)	287 (6.4) 1260 (40.4) 706 (16.4) 1673 (29.9)	31.851 (<0.001)
Monthly household income (1$ = 1000 KRW)	<1000 1000–1990 2000–2990 ≥3000	809 (16.4) 1300 (26.3) 964 (19.9)1683 (37.4)	410 (49.9) 286 (34.3) 82 (10.0) 52 (5.8)	399 (10.3) 1014 (24.8) 882 (21.7) 1631 (43.2)	308.36 (<0.001)
Employment status	Unemployed Employed	3631 (76.7) 1125 (23.3)	669 (81.2) 161 (18.8)	2962 (75.8) 964 (24.2)	10.28 (0.001)
Self-rated health status	Good Fair Poor	1322 (27.6) 1998 (42.7) 1436 (29.7)	177 (19.8) 333 (41.2) 320 (39.0)	1145 (29.1) 1665 (42.9) 1116 (28.0)	25.26 (<0.001)
Hypertension	No Yes	2070 (44.3) 2686 (55.7)	345 (40.7) 485 (59.3)	1725 (45.0) 2201 (55.0)	4.99 (0.026)
DM	No Yes	3761 (79.5) 995 (20.5)	666 (80.0) 164 (20.0)	3095 (79.4) 831 (20.6)	0.14 (0.708)
BMI		24.03 ± 0.05	24.10 ± 3.46	24.05 ± 3.24	0.411 (0.680)
Perceived stress	Low High	3946 (83.4) 808 (16.6)	689 (83.7) 141 (16.3)	3258 (83.3) 668 (16.7)	0.08 (0.778)
Perceived cognitive decline	No Yes	3284 (69.0) 1472 (31.0)	534 (63.3) 296 (36.7)	2750 (70.0) 1176 (30.0)	13.54 (<0.001)
Depressive symptoms	Not depressed Depressed (PHQ-9 ≥ 5)	3841 (80.5) 915 (19.5)	595 (71.5) 235 (28.5)	3246 (82.2) 680 (17.8)	46.45 (<0.001)
Regular exercise (more than 150 min/week)	Inactive Active	1332 (28.3) 3424 (71.7)	267 (32.6) 563 (67.4)	1065 (27.5) 2861 (72.5)	7.52 (0.006)
Current smoking	Non-smoker Smoker	4357 (91.2) 399 (8.8)	778 (93.8) 52 (6.2)	3579 (90.8) 347 (9.2)	6.16 (0.013)
Current alcohol drinking	Less than once a month More than once a month	3252 (66.8) 1504 (33.2)	645 (77.0) 185 (23.0)	2607 (65.0) 1319 (35.0)	41.06 (<0.001)

Note. No. of respondents is unweighted and percentage (%) is weighted; M ± SD = Mean and standard deviation, KRW: South Korean Won, DM: Diabetes Mellitus, BMI: Body Mass Index, PHQ-9: Patient Health Questionnaire.

**Table 2:** Comparison of sleep quality of Korean elders by living arrangements (*n* = 4756).

Sleep Quality	Living Alone (*n* = 830, 17.5%)	Living with Others (*n* = 3926, 82.5%)	Rao Scott χ^2^ or *t* (*p*)
Global PSQI score	6.47 ± 3.81	5.19 ± 3.38	8.942 (<0.001)
Global PSQI score > 6	435 (52.4)	1434 (36.5)	62.80 (<0.001)
PSQI components			
Sleep quality	744 (89.0)	3448 (87.6)	0.996 (0.319)
Sleep latency	570 (68.6)	2250 (56.7)	39.286 (<0.001)
Seep duration	563 (68.5)	2098 (54.8)	45.452 (<0.001)
Sleep efficiency	279 (33.6)	1117 (28.9)	7.106 (0.008)
Sleep disturbances	746 (90.6)	3444 (88.5)	2.909 (0.088)
Use of sleep pill	106 (12.3)	333 (8.2)	14.255 (<0.001)
Daytime dysfunction	310 (35.9)	1245 (31.5)	5.338 (0.021)

Values were presented as mean ± SD or n (%); No. of respondents is unweighted and percentage (%) is weighted; M ± SD: Mean and standard deviation; PSQI: Pittsburgh Sleep Quality Index, The PSQI component only presented the results of those who answered that they had sleep difficulties (More than 1 point of having difficulty in each of the PSQI components).

**Table 3:** Odds ratios (ORs) and 95% confidence intervals (CIs) for sleep quality by living arrangements (*n* = 4756).

Variables	Model 1		Model 2	
	OR (95% CI)	*p*	OR (95% CI)	*p*
Type of Living arrangement				
Living alone	1.300 (1.086–1.555)	0.004	1.276 (1.054–1.545)	0.013
Age ≥ 75	0.988 (0.861–1.133)	0.86	1.154 (0.992–1.342)	0.063
Gender (Female)	1.606 (1.400–1.843)	<0.001	1.629 (1.382–1.920)	<0.001
Education level				
≥High school	1 (Reference)		1 (Reference)	
Middle school	1.242 (1.033–1.493)	0.021	1.161 (0.949–1.421)	0.146
≤Elementary school	1.465 (1.238–1733)	<0.001	1.213 (1.011–1.457)	0.038
Illiteracy	1.496 (1.157–1.935)	0.002	1.233 (0.925–1.642)	0.152
Monthly household income				
≥3000	1 (Reference)		1 (Reference)	
2000–2990	1.060 (0.893–1.259)	0.504	1.067 (0.886–1.285)	0.495
1000–1990	1.051 (0.883–1.250)	0.577	1.057 (0.880–1.270)	0.554
<1000	1.459 (1.177–1.809)	0.001	1.155 (0.915–1.458)	0.225
Employment status				
Unemployed	0.790 (0.672–0.929)	0.004	1.121 (0.945–1.331)	0.19
Self-rated health status				
Good			1 (Reference)	
Fair			1.371 (1.154–1.636)	<0.001
Poor			1.989 (1.616–2.448)	<0.001
Current smoking (Smoker)			0.882 (0.685–1.136)	0.332
Alcohol drinking (More than once a month)			0.848 (0.721–0.998)	0.047
Regular exercise (Inactive)			1.278 (1.099–1.486)	0.001
Perceived cognitive decline (Yes)			1.534 (1.330–1.771)	<0.001
Hypertension (Yes)			1.031 (0.902–1.178)	0.656
Depressive symptoms (Yes)			4.616 (3.845–5.543)	<0.001

No. of respondents is unweighted. Model 1: adjusted for age, gender, education level, monthly household income and employment status. Model 2: further adjusted for current smoking, alcohol drinking, regular exercise, self-rated health status, perceived cognitive decline, hypertension and depressive symptoms.

**Table 4:** Adjusted odds ratios (ORs) and 95% confidence intervals (CIs) for sleep quality according to living arrangements by gender (*n* = 4756).

Gender	Type of Living Arrangement	Crude		Model 1		Model 2	
		OR (95% CI)	*p*	OR (95% CI)	*p*	OR (95% CI)	*p*
Male (*n* = 2029)	Living with others	1 (Reference)		1 (Reference)		1 (Reference)	
	Living alone	1.595 (1.160–2.192)	0.004	1.208 (0.837–1.744)	0.312	1.089 (0.729–1.628)	0.677
Female (*n* = 2727)	Living with others	1 (Reference)		1 (Reference)		1 (Reference)	
	Living alone	1.542 (1.293–1.840)	<0.001	1.353 (1.099–1.666)	0.004	1.359 (1.088–1.696)	0.007

No. of respondents is unweighted. Model 1: adjusted for age, education level, monthly household income and employment status. Model 2: further adjusted for current smoking, alcohol drinking, regular exercise, self-rated health status, perceived cognitive decline, hypertension and depressive symptoms.

## Data Availability

The data presented in this study are available on request from the fist author.
